# Practice activity trends among oral and maxillofacial surgeons in Australia

**DOI:** 10.1186/1472-6963-4-37

**Published:** 2004-12-21

**Authors:** David S Brennan, A John Spencer, Kiran A Singh, Dana N Teusner, Alastair N Goss

**Affiliations:** 1 Australian Research Centre for Population Oral Health, Dental School, Faculty of Health Sciences, The University of Adelaide, Australia; 2 Oral and Maxillofacial Surgery Unit, Dental School, Faculty of Health Sciences, The University of Adelaide, Australia

## Abstract

**Background:**

The aim of this study was to describe practice activity trends among oral and maxillofacial surgeons in Australia over time.

**Methods:**

All registered oral and maxillofacial surgeons in Australia were surveyed in 1990 and 2000 using mailed self-complete questionnaires.

**Results:**

Data were available from 79 surgeons from 1990 (response rate = 73.8%) and 116 surgeons from 2000 (response rate = 65.1%). The rate of provision of services per visit changed over time with increased rates observed overall (from 1.43 ± 0.05 services per visit in 1990 to 1.66 ± 0.06 services per visit in 2000), reflecting increases in pathology and reconstructive surgery. No change over time was observed in the provision of services per year (4,521 ± 286 services per year in 1990 and 4,503 ± 367 services per year in 2000). Time devoted to work showed no significant change over time (1,682 ± 75 hours per year in 1990 and 1,681 ± 94 hours per year in 2000), while the number of visits per week declined (70 ± 4 visits per week in 1990 to 58 ± 4 visits per week in 2000).

**Conclusions:**

The apparent stability in the volume of services provided per year reflected a counterbalancing of increased services provided per visit and a decrease in the number of visits supplied.

## Background

In Australia the majority of dentists work in private general practice [1]. Relatively few are specialists (10.8%), of which 16.8% are oral and maxillofacial surgeons accounting for 1.9% of all practising dentists. The major trends in oral health in Australia over recent decades indicate improved oral health among the population. For example, there has been a dramatic decline in the percentage of edentulous adults [2,3], and caries experience among children has declined since the 1970s [4], although in the later half of the 1990s improvements in child oral health had ceased [5]. Service trends in private general practice have reflected the trends towards improved oral health with a shift towards higher provision of services such as diagnostic, preventive and endodontic consistent with the retention and maintenance of a natural dentition [6]. Practice activity patterns among private general practitioners have shown declining levels of visits supplied but stable levels of time devoted to work [7].

Identifying trends over time in the practice activity of oral and maxillofacial surgeons is a key element in planning by informing debate on issues relevant to the speciality such as the future supply of services and training needs. Previous Australian data have shown the distribution of oral and maxillofacial surgery was dominated by dentoalveolar services [8]. However, the dominance of dentoalveolar surgery might be reduced if the practice patterns of surgeons in relation to third molar surgery were influenced by debate on the development of standards and criteria of care [9], and ongoing assessment of the risks and benefits of removal of third molars [10]. While the core of oral and maxillofacial surgery is dentoalveolar, the knowledge of the orofacial region forms the basis for the wider scope of the modern specialty [11]. Since 1996 it has been mandatory that all trainees in Australia enter dual degree programs and then exit with the College fellowship, as a result the percentage of medically qualified surgeons has increased from 2.5% to 17.1% between 1990 and 2000 [12]. It has been reported that oral and maxillofacial surgeons with medical qualifications, while maintaining a broad scope, tended to have a greater range of procedures within the major groupings [13].

Considering the trend towards oral and maxillofacial surgeons gaining medical qualifications and the potential impact that this may have on practice activity, the aims of this study are to describe practice activity trends among oral and maxillofacial surgeons in Australia in 1990 and 2000 in terms of time worked, visits supplied and services provided.

## Methods

### Study design/sample

All registered oral and maxillofacial surgeons in Australia were surveyed in both 1990 and 2000. A surgeon must be registered with a dental board in the state/territory in which they practice. For the purposes of this analysis trainees were excluded. Although some of the same surgeons may have responded to the survey at both points in time for this study the design and analysis is treated as two sequential cross-sectional surveys.

### Data collection and analyses

Data were collected using mailed self-complete questionnaires with a primary approach letter sent initially to each surgeon, followed a week later by the survey materials, with a reminder card two weeks later, and up to four follow-up mailings of survey materials to surgeons who had not yet responded in order to ensure higher response rates [14]. Surgeon background characteristics and practice factors were described using percentages and compared using chi-square tests for 1990 and 2000. Service rates, time devoted to work and number of visits supplied by surgeons were described using means and compared between 1990 and 2000 using general linear models. All analyses were performed using SAS software [15].

### Study variables

**Figure 1:**
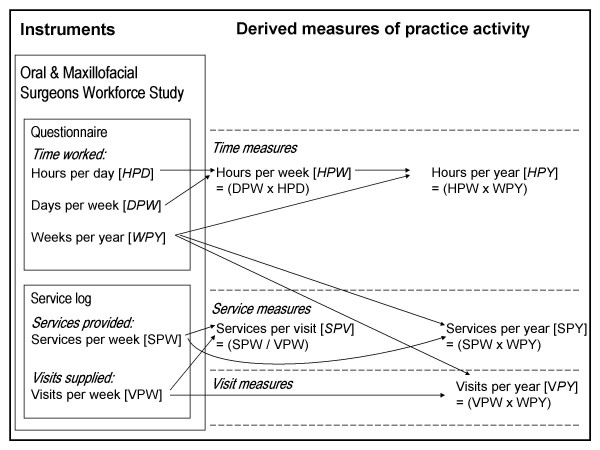
Derivation of practice activity measures

The questionnaire was designed to provide comprehensive coverage of a range of workforce issues and the analysis presented here is limited to a subset of the total number of variables that were collected. The questionnaire included surgeon demographic and background variables (e.g., year of birth, sex, place of birth, qualifications,), practice details (e.g., level of activity in private and public practice, type of practice, level of practice activity), and a log of services provided in a typical week. Surgeons recorded details of the patients they treated over a one-week period. Main areas of service were classified as dentoalveolar, trauma, pathology, orthognathic, reconstructive surgery and other/major medical compromise. An outline of the key variables collected and how measures of time worked, services provided and visits supplied were derived is presented in Figure 1. The time measures of hours per day, days per week and weeks per year worked were used to calculate hours per week and hours per year worked, and were used along with visits per week and services per week to calculate visits per year and both services per visit and services per year.

## Results

### Response and background characteristics by year of study

Data were available from 79 surgeons from 1990 (response rate = 73.8%) and 116 from 2000 (response rate = 65.1%). Service provision data were available for 4,847 patients from 1990 and 3,292 patients from 2000. Table 1 shows the majority of surgeons in both 1990 and 2000 were in the age group 40–49 years, were male and born in Australia. Similarly, in both 1990 and 2000 the majority of surgeons worked 80+% in private practice. The only significant difference between 1990 and 2000 in Table 1 was the higher percentage of surgeons with dual qualifications, having a medical degree and College fellowship FRACDS (OMS) in addition to a dental degree.

### Service rates

The rate of provision of services is presented in Table 2. The distribution of services per visit was dominated by dentoalveolar services in both 1990 and 2000. The overall rate of services per visit increased between 1990 and 2000, reflecting increased rates of pathology and reconstructive surgery. The distribution of services per year reflected the pattern for services per visit with dentoalveolar services dominating. However, there were no significant differences over time in the rate of provision of services per year.

### Practice activity

**Figure 2:**
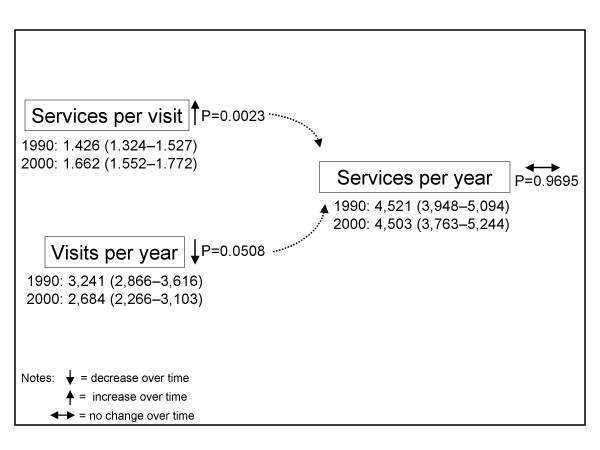
Relationship of service and visit measures. Data show mean (95% confidence interval).

The number of hours per year devoted to work by surgeons did not change significantly between 1990 and 2000, reflecting stable levels of hours per day, days per week and weeks per year worked. However, the number of visits per week supplied by surgeons decreased between 1990 and 2000. The number of visits per year supplied by surgeons also showed a trend towards a decreased number of visits over time, but the change (P = 0.0508) was not significant at the P < 0.05 level. The relationship between the service and visit measures is presented in Figure 2, which shows that the stability in the number of services provided per year involved a counterbalancing of increased rates of service per visit and decreased numbers of visits supplied.

## Discussion

While the findings of this study are based on a small sample size this primarily reflects the size of the population studied. Oral and maxillofacial surgeons comprise a small percentage of the dental workforce [1], hence all registered surgeons were included in order to maximise the sample size available for analysis. While smaller samples can reduce statistical power it is still possible to detect significant differences when the effect size is sufficiently large, and a number of statistically significant differences were observed. While small sample sizes can sometimes result in bias, the achievement of adequate response rates in this study did not suggest response bias issues were likely [16]. The use of a sequential cross-sectional design while unable to address change over time at an individual level, as in a longitudinal design that traces the same subjects, has the advantage of being representative at both points by the inclusion of new subjects that have entered the study population (assuming no bias has been introduced through other means).

The practice patterns of oral and maxillofacial surgeons have been reported to be largely stable, showing no change between 1990 and 2000 in their age and sex distribution, place of birth, practice activity level, referral sources, mix of cases and perceptions of work [12]. The distribution of main areas of service has also remained relatively stable over time [17]. However, there were signs of change in the increased percentage of dual qualified surgeons, and changes in rates of some non-dentoalveolar surgical procedures over the course of the study. While still a minority of surgeons, those with dual qualifications had a different service profile with higher rates of orthognathic surgery, dental implants, and bone graft procedures. While the mix of cases was dominated by dentoalveolar rather than major maxillofacial surgery in both 1990 and 2000, there appears to be a beginning of an expansion of some selected non-dentoalveolar surgical procedures. However, the stability in orthognathic surgery rates per year observed here indicates that the higher odds of orthognathic surgery among dual qualified surgeons [18] has not increased the total provision of this type of surgery.

The decline in patient visits supplied by oral and maxillofacial surgeons, while statistically significant for visits supplied per week, was not statistically significant at the conventional level of P < 0.05 for visits supplied per year. This partly reflects the stability in weeks per year worked, one of the component measures used to derive visits per year. However, the number of visits per year was at the borderline of statistical significance (P = 0.0508) and considerations other than a reliance on P values is recommended in the literature [19], with more emphasis on estimation through the use of confidence intervals to accompany point estimates [20]. A key guide is the measure of effect size, or the size of the difference being reported, which can be factored into considerations of clinical significance at the individual level and public health importance when aggregated across the individuals making up a population.

The trends observed in practice activity among oral and maxillofacial surgeons show parallels with private general practice dentists in Australia. Trends in private general practice have also shown a counterbalancing effect of declining numbers of visits supplied with increased rates of services per visit resulting in a stable level of provision of services per year. Medical general practitioners have shown a trend towards longer consultations [21], consistent with the trend observed for the dental labour force. Despite the divergence in length and scope of training that oral and maxillofacial surgeons are required to fulfil, the convergence in practice activity trends may indicate specific health labour force or even broader labour force issues influencing both groups similarly. The Australian health and community services labour force in general has shown a trend towards working less hours per year with an increase in the percentage working part-time between 1996 and 2002 [22], which is different to the stable number of hours per year worked by both dentists and oral and maxillofacial surgeons. However, the reported decline in hours worked per week for medical specialists brings their average clinical hours (41.5 hours per week) close to 39.5 hours per week reported for oral and maxillofacial surgeons [23].

Possible explanations for the lower levels of patient visits per hour among private general practice dentists include increased numbers of older patients [24], who may have complex treatment needs which require more services or take longer to complete. Changes have been observed in the distribution of the characteristics of patients treated and visits supplied by oral and maxillofacial surgeons over the study period [17], with the most marked difference being a shift to an older age distribution of patients consistent with demographic trends projecting an increase in middle-aged and older adults in the Australian population. Data from New Zealand have shown increases in the rate and number of injuries among older people and a general increase in the contribution of falls to the occurrence of trauma [25]. It may also be that as Australians retain more teeth into older age trauma services will expand in these age groups of patients. However, most trauma treated by oral and maxillofacial surgeons is related to the jaw rather than teeth and despite the increased percentage of patients aged 45 years and older, the majority of patients treated by oral and maxillofacial surgeons remained in the age groups 18–24 and 25–44 years.

Historical records have indicated that average length of dental appointments changed little over the period 1960–61 to 1974–75, but there was an increase since 1974–75 that was quite marked across the 1977–78 to 1982–83 period [26-29], and continued to increase through to 2001 [30]. Cross-infection control procedures may be another possible source of influence on productivity associated with either increased appointment or change-over times. The operation of such effects on productivity has implications for planning the delivery of services.

## Conclusions

Estimates of the capacity to supply services and projections of labour force requirements need to consider that while the rate of services per visit has increased this has been counterbalanced by decreases in the number of visits supplied. This has resulted in a stable volume of services provided by oral and maxillofacial surgeons per year over the study period.

## Competing interests

The author(s) declare that they have no competing interests.

## Authors' contributions

DSB performed data analyses and drafting of the manuscript. AJS provided overall supervision of the project. KAS performed data processing and preliminary analyses. DNT was involved in data collection. ANG provided specialist advice on oral and maxillofacial surgery. All authors contributed to and approved the manuscript.

## Pre-publication history

The pre-publication history for this paper can be accessed here:



## Figures and Tables

**Table 1:** Distribution of background and practice characteristics

	**1990**	**2000**
**Age of surgeon^ns^**	%	%
20–29 years	1.3	0.0
30–39 years	27.9	14.6
40–49 years	39.2	36.6
50–59 years	19.0	34.2
60+ years	12.7	14.6
		
**Sex of surgeon^ns^**		
Male	94.9	91.3
		
**Place of birth^ns^**		
Australia	72.4	67.1
		
**Practice activity level^ns^**		
80+% private	64.1	71.4
		
Qualifications **		
Dental plus medical degree & FRACDS (OMS)	2.5	17.1

**(P < 0.01), ns (not statistically significant) χ^2 ^test

**Table 2:** Rate of provision of services by time of study

	**Services per visit**					**Services per year**				
		
	**1990**		**2000**			**1990**		**2000**		
		
	**Mean**	**(SE)**	**Mean**	**(SE)**	**P**	**Mean**	**(SE)**	**Mean**	**(SE)**	**P**
Dentoalveolar	1.070	(.065)	1.258	(.073)	0.0603	3400	(265)	3397	(318)	0.9937
Trauma	0.073	(.014)	0.046	(.010)	0.1593	239	(44)	145	(36)	0.1176
Pathology	0.096	(.012)	0.141	(.021)	*0.0490	289	(36)	374	(57)	0.1858
Orthognathic	0.073	(.014)	0.076	(.016)	0.8745	260	(51)	220	(48)	0.5756
Reconstructive	0.059	(.009)	0.099	(.014)	*0.0178	226	(41)	272	(46)	0.4528
										
Total services	1.425	(.051)	1.662	(.055)	**0.0023	4521	(286)	4503	(367)	0.9695

*(P < 0.05), **(P < 0.01) GLM

**Table 3:** Time and visit measures by time of study

	**1990**		**2000**		
	
	**Mean**	**(SE)**	**Mean**	**(SE)**	**P**
**Time measures**					
Hours per day	7.69	(0.23)	7.84	(0.34)	0.7071
Days per week	4.62	(0.12)	5.04	(0.28)	0.1378
Weeks per year	46.35	(0.48)	46.65	(0.70)	0.7181
Hours per year	1682.50	(75.37)	1681.17	(94.91)	0.9912
					
**Visit measures**					
Visits per week	69.80	(3.94)	57.85	(4.28)	*0.0455
Visits per year	3241.31	(187.62)	2684.24	(207.88)	0.0508

*(P < 0.05) GLM
